# Effect of Particulate Matter 2.5 on Fetal Growth in Male and Preterm Infants through Oxidative Stress

**DOI:** 10.3390/antiox12111916

**Published:** 2023-10-26

**Authors:** Sunwha Park, Eunjin Kwon, Gain Lee, Young-Ah You, Soo Min Kim, Young Min Hur, Sooyoung Jung, Yongho Jee, Mi Hye Park, Sung Hun Na, Young-Han Kim, Geum Joon Cho, Jin-Gon Bae, Soo-Jeong Lee, Sun Hwa Lee, Young Ju Kim

**Affiliations:** 1 Department of Obstetrics and Gynecology, College of Medicine, Ewha Womans University, Seoul 07985, Republic of Korea; clarrissa15@gmail.com (S.P.); yerang02@naver.com (Y.-A.Y.); k0507hym@hanmail.net (Y.M.H.); jsmed9006@naver.com (S.J.); 2 Division of Allergy and Respiratory Disease Research, Department of Chronic Disease Convergence Research, Korea National Institute of Health, Cheongju-si 28159, Republic of Korea; friendkej1004@hanmail.net; 3 Graduate Program in System Health Science and Engineering, Ewha Womans University, Seoul 07985, Republic of Korea; loveleee0102@gmail.com (G.L.); soomnium@naver.com (S.M.K.); 4 Advanced Biomedical Research Institute, Ewha Womans University Seoul Hospital, Seoul 07804, Republic of Korea; jyongho@ewha.ac.kr; 5 Department of Obstetrics and Gynecology, Ewha Womans University Seoul Hospital, Seoul 07804, Republic of Korea; ewhapmh@ewha.ac.kr; 6 Department of Obstetrics and Gynecology, School of Medicine, Kangwon National University, Chuncheon-si 24289, Republic of Korea; lahun@kangwon.ac.kr; 7 Department of Obstetrics and Gynecology, College of Medicine, Yonsei University, Seoul 03722, Republic of Korea; yhkim522@yuhs.ac; 8 Department of Obstetrics and Gynecology, College of Medicine, Korea University, Seoul 02841, Republic of Korea; geumjoon@korea.ac.kr; 9 Department of Obstetrics and Gynecology, School of Medicine, Keimyung University, Dongsan Medical Center, Daegu 42601, Republic of Korea; gonmd@dsmc.or.kr; 10 Department of Obstetrics and Gynecology, College of Medicine, Ulsan University, Ulsan 44610, Republic of Korea; exsjlee@uuh.ulsan.kr; 11 Seegene Medical Foundation, Seoul 04805, Republic of Korea; lshkim@neolab.co.kr

**Keywords:** 8-hydroxy-2-deoxyguanosine, biomarker, indoor air, low birth weight, particulate matter, preterm birth, sex difference, small for gestational age

## Abstract

Particulate matter 2.5 (PM_2.5_) levels are associated with adverse pregnancy outcomes. In this retrospective cohort study, we examined whether the concentration of indoor PM_2.5_ affected pregnancy outcomes. Additionally, we evaluated biomarkers of pregnancy-related complications caused by fine dust. We collected clinical information and data based on residential addresses from the Air Korea database to assess PM_2.5_ exposure levels. As a multicenter prospective cohort study, we measured the indoor PM_2.5_ concentration and inflammatory and oxidative stress markers. The PM_2.5_ concentration of the low-birth-weight (LBW) delivery group was 27.21 μg/m^3^, which was significantly higher than that of the normal-birth-weight (NBW) group (26.23 μg/m^3^) (*p* = 0.02). When the newborns were divided by sex, the PM_2.5_ concentration of the LBW group was 27.89 μg/m^3^ in male infants, which was significantly higher than that of the NBW group (26.26 μg/m^3^) (*p* = 0.01). In the prospective study, 8-hydroxy-2-deoxyguanosine significantly increased in the high-concentration group (113.55 ng/mL, compared with 92.20 ng/mL in the low-concentration group); in the high-concentration group, the rates of preterm birth (PTB) and small size for gestational age significantly increased (*p* < 0.01, *p* = 0.01). This study showed an association between PM_2.5_, oxidative stress, and fetal growth, with the PTB group being more vulnerable.

## 1. Introduction

Low birth weight (LBW; birth weight < 2500 g, regardless of gestational age), small for gestational age (SGA; birth weight below the 10th percentile for gestational age), and preterm birth (PTB; delivery at <37 weeks of gestation) are complications of pregnancy that directly affect the prognosis of newborns [1,2,3,4,5,6,7]. The incidence of LBW varies by country (range, 6–25%); however, LBW births are associated with short- and long-term complications [8,9]. The prevalence of SGA births is approximately twice that of LBW [10]. Previous studies have investigated sociodemographic and medical risk factors as well as environmental risks (i.e., exposure to toxic substances) for LBW [10]. 

Particulate matter (PM) 2.5 (PM_2.5_, particles with an aerodynamic diameter of ≤2.5 μm), one of the major air pollutants, has been reported to be associated with various adverse pregnancy outcomes [11,12]. In particular, associations between PM_2.5_ and LBW, PTB, and SGA infants have been reported [5,6,7,11]. Most studies have conducted exposure assessments using outdoor air quality measurements [5,7,11,13]. Recently, it was found that 80–90% of individuals live indoors, and the importance of indoor air quality has been gradually emphasized [14,15]. Therefore, the importance of measuring indoor PM concentrations is increasing, and individual indoor fine dust exposure may reveal better causal relationships between PM_2.5_ and pregnancy outcomes [14,16,17]. 

There is a lack of understanding of the pathogenesis of PM_2.5_ affecting fetal growth. Various studies have been conducted to understand the cause of fetal growth restriction [18,19]. The analysis of sex differences is useful in understanding biological mechanisms. Although biological mechanisms are influenced by PM regardless of sex, sex-specific effects remain controversial [11,16,20]. It is also controversial whether exposure to PM at any stage of pregnancy has a greater effect on fetal growth [21,22]. 

Recently, various biomarkers have been developed to evaluate exposure to fine dust [23], and attempts have been made to explain fetal growth based on DNA methylation or telomere length in cord blood [16,24,25,26]. However, cord blood is not suitable for biomonitoring, because it is difficult to sample during pregnancy. Although there are many studies on oxidative stress, inflammation, DNA damage, and epigenetic modulation as biomarkers of exposure to PM, there are few biomarker studies on pregnant women [23]. 

Therefore, this study aimed to examine whether the concentration of PM_2.5_ affects fetal growth through indoor PM measurement. We also evaluated biomarkers of pregnancy-related complications caused by fine dust as well as indicators for the biomonitoring of PM_2.5_ exposure. 

## 2. Materials and Methods 

### 2.1. Study Design of Cohort I 

The first study was a hospital-based retrospective cohort study of 1880 pregnant women who delivered live babies between 2010 and 2015 at the Ewha Womans University Mokdong Hospital (EUMC 2020-07-043). We collected information on maternal age; body mass index (BMI); gestational age at birth (GAB); neonatal sex; weight and height at birth; appearance, pulse, grimace, activity, and respiration (APGAR) score; and place of residence at the time of delivery. The Air Korea database was used for PM_2.5_ exposure assessment, which includes hourly accumulated air pollution monitoring data for components such as sulfur dioxide, PM_10_, carbon monoxide, nitrogen dioxide, and ozone from the Ministry of Environment of Korea. The level of PM_2.5_ exposure was measured using the Community Multiscale Air Quality (CMAQ) modeling system. PM_2.5_, from CMAQ modeling data, was estimated using meteorological research and forecasting models comprising three overlapping weather data sources at 3, 9, and 27 km for a specific time period. PM_2.5_ exposure levels during pregnancy were determined based on residential address (city, county, and district, or si, gun, and gu in the Korean language). For each address, daily PM_2.5_ concentrations were matched to each individual according to their delivery date. Each individual’s outdoor air quality data were collected for each pregnancy trimester, extending to data from the week of delivery. PM_2.5_ data were collected from 2009 to 2015, depending on the pregnancy period of the study participants. 

**Figure 1:**
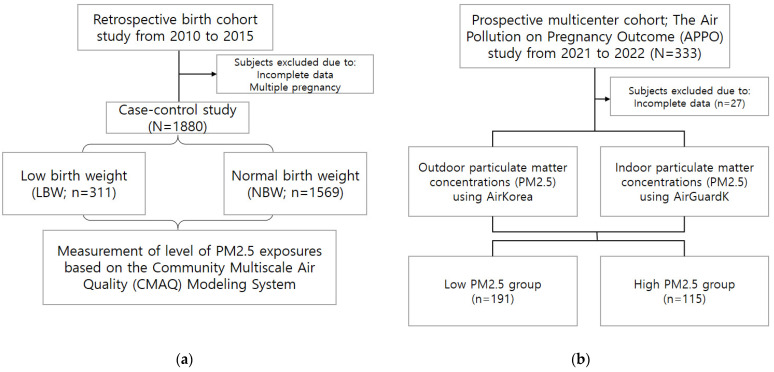
Study flow chart of Cohorts I (**a**) and II (**b**).

We stratified the groups by birth weight and height and performed Student’s *t*-test (Figure 1a). LBW was defined as a weight at birth of <2500 g, and low birth height (LBH) was defined as a height at birth of <46.3 cm for male and <45.6 cm for female infants. 

### 2.2. Study Design of Cohort II 

A multicenter prospective cohort study of air pollution in pregnant women (APPO) was conducted to investigate the effects of PM on mothers and fetuses by recruiting > 1200 participants between January 2021 and December 2023 in seven university hospitals across Korea. Each hospital was located in a metropolitan area, an industrial complex, or a mountainous area. The participants were singleton pregnant women with no underlying diseases before 28 weeks of gestation. 

In the second study, 333 women who had delivered were selected as participants (Figure 1b). We collected data on basic demographic and health-related characteristics including age, BMI, socioeconomic status, and obstetric history. Routine blood tests were conducted to measure the white blood cell (WBC) counts and high-sensitivity C-reactive protein (hs-CRP) levels as inflammatory markers, and urine samples were collected during the second trimester. The pregnancy outcomes were evaluated after delivery. 

This study was approved by the Ethical Research Committee of Ewha Womans University Mokdong Hospital (EUMC 2021-04-032), Yonsei University Severance Hospital (4-2021-0414), Kangwon National University Hospital (KNUH-B-2021-04-012-008), Keimyung University Dongsan Medical Center (2021-04-073), Korea University Guro Hospital (2021GR0233), Ewha Womans University Seoul Hospital (2021-04-022), and Ulsan University Hospital (2022-04-020). All participants provided written informed consent.

### 2.3. PM Exposure Assessment 

The indoor PM_2.5_ concentration was measured by placing a fine dust meter at breathing height in the living room of a pregnant woman’s house. An AirGuard K (Kweather Co., Seoul, Republic of Korea) instrument was used for measurements using the sensor-based method. The indoor PM_2.5_ was measured online at 1-min intervals. The measured indoor PM_2.5_ data were stored in an indoor air quality monitoring platform (IAQ Station) using Long-Term Evolution. Measurements were performed for at least 1 week, and the measured concentration values were recorded in real time using the Internet of Things and Information and Communication Technology. For indoor PM_2.5_, after removing outliers, the average value of measurements for each trimester of pregnancy was derived, and the average of the two or three trimesters was defined as PM_2.5_. The participants were divided according to the PM_2.5_ exposure level as follows: <10 µg/m^3^ (low PM_2.5_ group) and ≥10 µg/m^3^ (high PM_2.5_ group) concentration groups, which are the World Health Organization’s annual limit standard concentrations (2005 air quality guidelines).

Outdoor PM_2.5_ concentrations were collected from a nearby urban atmospheric measurement network based on the residential addresses of the study participants. The Urban Air Monitoring Station data used in this study were obtained from the Air Korea database (https://www.airkorea.or.kr/web (accessed on 1 May 2021)) of the Korean Ministry of the Environment. 

### 2.4. Collection of Blood and Urine Samples

We collected 5 mL maternal venous blood and 15 mL urine. Blood and urine samples were collected at regular follow-up visits. Whole blood samples collected in ethylenediaminetetraacetic acid tubes were transferred to cryotubes, and urine was stored in one cryotube. Samples were transferred to the institution (the Seegene Medical Foundation, Seoul, Republic of Korea) on the same day as collection under refrigeration to prevent deterioration (−80 °C). 

### 2.5. Measurement of Oxidative Stress and Inflammatory Markers 

The oxidative stress markers 8-hydroxy-2-deoxyguanosine (8-OHdG) and malondialdehyde (MDA) were measured in urine samples collected during the second trimester of pregnancy. The collected urine samples were transported directly to the laboratory and stored at −80 °C before processing. The urine samples were filtered with a 0.2-µm filter (Sartorius, S6534-FMOSK, Göttingen, Germany) for the assay and diluted at 1:20 to measure the concentration of 8-OHdG using enzyme-linked immunosorbent assay (ELISA) kits (Abcam, Ab201734, Waltham, Boston, MA, USA). The concentration of MDA in urine was measured using an MDA ELISA kit (ab118970; Abcam). Among the inflammatory markers, the WBC count was measured using an XN-9000 (Symex, Kobe, Japan) according to the manufacturer’s protocol, from the participant’s whole blood sample, through an automated complete blood cell count. The hs-CRP level was measured using a particle-enhanced immunoturbidimetric assay according to the manufacturer’s protocol using a Cobas 8000 C702 analyzer (Roche, Basel, Switzerland). 

### 2.6. Statistical Analysis 

Clinical characteristics were analyzed according to continuous variables (age and BMI) and categorical variables (marital status, education level, occupation, monthly income, gravidity, and pregnancy methods). Pregnancy outcomes were analyzed according to continuous (GAB, birth weight, height, and APGAR score) and categorical variables (delivery mode, neonate sex, and pregnancy complications). Pregnancy complications were defined using the following criteria (LBW: birth weight of <2500 g; LBH: birth height <46.3 cm for male and <45.6 cm for female infants; SGA: birth weight < 10th percentile for gestational age; PTB: delivery at <37 weeks of gestation) and analyzed as categorical variables. Oxidative stress (8-OHdG and MDA) and inflammatory markers (hs-CRP and WBC count) were analyzed as continuous variables. Categorical variables were expressed as frequencies (percentages) and analyzed using chi-square and Fisher’s exact tests. Continuous variables were expressed as means ± standard deviations (SDs) and were compared using the *t*-test or Mann–Whitney U test. Logistic regression model was used to estimate the associations between LBW and PM_2.5_ exposure (1 μg/m^3^) in each trimester with adjustment for covariates (maternal age, parity, maternal pre-pregnancy BMI, preeclampsia, and gestational age). Statistical significance was defined as *p* < 0.05. All statistical analyses were performed using the Statistical Package for the Social Sciences (version 20.0; IBM Corp., Armonk, NY, USA). 

## 3. Results 

### 3.1. Association between PM_2.5_ Exposure and Birth Weight and Height According to Neonatal Sex Using a Retrospective Cohort Study

Among the 1880 neonates, 1569 had a normal birth weight (NBW) and 311 had LBW. The two groups showed significant differences in GAB, birth weight, height, and APGAR scores (*p* < 0.01); however, there were no significant differences in maternal age, pre-pregnancy BMI, and neonatal sex (Table 1). The average outdoor PM_2.5_ concentration was 27.35 µg/m^3^ (Table 2 and Supplementary Table S1). The PM_2.5_ concentration in the second trimester for pregnant women who delivered LBW babies was 27.21 µg/m^3^, which was significantly higher than the value of 26.23 µg/m^3^ in the NBW group (*p* = 0.02, Table 3). When the delivered newborns were divided by sex, the PM_2.5_ concentration in the second trimester for male LBW babies was 27.89 µg/m^3^, which was significantly higher than the value of 26.26 µg/m^3^ in male infants with NBW (*p* = 0.01), but there was no significant difference in female infants (*p* = 0.74, Table 3).

When the neonates were divided into term and PTB groups, the concentration of PM_2.5_ for the LBW infants in the PTB group was 27.29 ug/m^3^, which was significantly higher than the concentration of 25.89 µg/m^3^ for NBW babies, respectively (*p* = 0.02, Table 4). In the PTB group, the PM_2.5_ concentration of the preterm normal birth weight (PNBW) and preterm low birth weight (PLBW) groups showed significant differences between the first and second trimesters, respectively (*p* = 0.05, *p* = 0.04), with more significant differences in the male group (*p* < 0.01, *p* = 0.01, Table 4). In contrast, in female infants in the PTB group, there were no statistically significant differences between the PNBW and PLBW groups in any trimester (*p* = 0.88, Table 4). In the term birth group, there was no significant difference between the NBW and LBW groups at term in any trimester (*p* = 0.30; Supplementary Table S2).

Pregnant women who were exposed to PM_2.5_ had a significantly higher risk of LBW than those who were not exposed, with an adjusted odds ratio (OR) of 1.06 (95% confidence interval [CI]: 1.01–1.10, Table 5). In male infants, the risk of LBW was higher, with an OR of 1.12. The susceptibility period was in the first and second trimester, with ORs of 1.05 and 1.07, respectively (95% CI: 1.01–1.10 and 1.03–1.12, Table 5).

### 3.2. Study Population of APPO and Measurement and Correlation of Indoor/Outdoor PM_2.5_

An analysis was performed on 306 of 333 delivery participants, excluding those with incomplete data. According to the PM_2.5_ exposure levels of the participants, the numbers in the low and high PM_2.5_ groups were 191 and 115, respectively (Table 6). There were no significant differences in the characteristics of the study population between the high and low PM_2.5_ concentration groups (Table 6).

**Figure 2:**
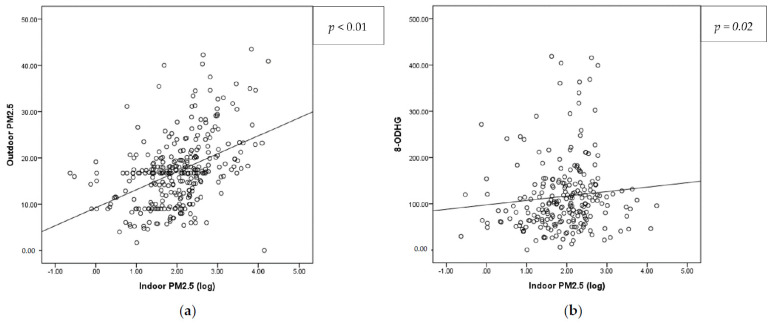
Correlation analysis between (**a**) indoor and outdoor PM_2.5_, and (**b**) indoor PM_2.5_ and 8-OHdG. 8-OHdG, 8-hydroxy-2-deoxyguanosine; PM_2.5_, particulate matter 2.5.

The average of the indoor PM_2.5_ concentration in the second trimester of pregnancy among the APPO study participants was 10.57 µg/m^3^, and that of the outdoor PM_2.5_ was 17.27 µg/m^3^. The two measurements showed a statistically significant positive correlation (*p* < 0.01, r^2^ = 0.187) (Table 2, Figure 2a). The concentration of outdoor PM_2.5_ in Cohort I was significantly lower than that in Cohort II (*p* < 0.01, Table 2). 

### 3.3. Association between PM_2.5_ Exposure and Pregnancy Complications 

**Figure 3:**
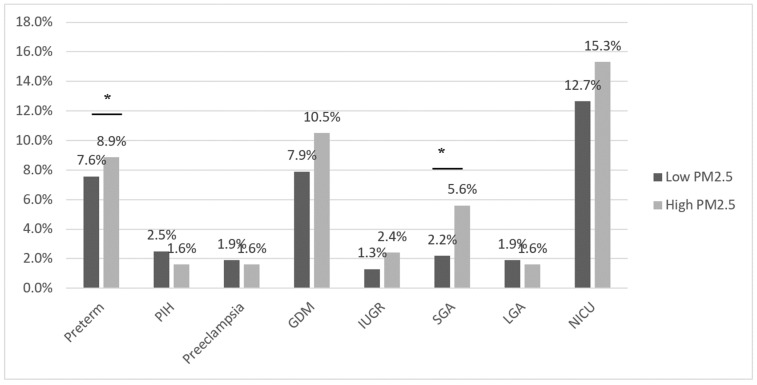
Pregnancy complications in the PM_2.5_ concentration group. GDM, gestational diabetes mellitus; IUGR, intrauterine growth restriction; LGA, large for gestational age; NICU, neonatal intensive care unit; PIH, pregnancy-induced hypertension; PM_2.5_, particulate matter 2.5; SGA, small for gestational age. * *p* < 0.05 considered statistically significant.

The oxidative stress marker 8-OHdG was significantly increased in the high-concentration group to 113.55 ng/mL, compared to 92.20 ng/mL in the low-concentration group, and there was a positive correlation (*p* = 0.02, r^2^ = 0.010) (Table 7, Figure 2b). There were no differences in the MDA or inflammatory marker levels between the two groups (Table 7). There were no differences in GAB, delivery mode, neonatal sex, birth weight, or APGAR score (Table 8). The mean birth height was 48.9 cm in the high-concentration group, which was significantly lower than that in the low-concentration group (49.5 cm; *p* = 0.04; Table 8). In the high-concentration group, PTB significantly increased from 4.2% to 13.2%, and SGA significantly increased from 0.6% to 5.1% (*p* < 0.01, *p* = 0.01) (Table 8, Figure 3).

## 4. Discussion

In this study, the association between PM_2.5_ and LBW was confirmed through a retrospective cohort study, and its effect was found to be more pronounced in the male and PTB groups. Furthermore, a multicenter prospective cohort study revealed the relationship between PM_2.5_ and birth height, SGA, and PTB by measuring the actual indoor PM_2.5_. The oxidative stress marker 8-OHdG, which was used to explore the pathogenesis of these pregnancy complications and identify biomarkers, showed a positive correlation with PM_2.5_. 

Our results are similar to those of previous studies that showed an association between PM_2.5_ and fetal growth and PTB [11,27,28,29,30]. Regarding the biological mechanism by which PM_2.5_ affects fetal growth, the effects of oxidative stress, placental inflammation or dysfunction, endothelial dysfunction, and blood coagulation have been reported [18,19]. The pathogenesis of PM-induced PTB involves systemic inflammation caused by the inhalation of toxic particles [31,32]. Many large-scale studies have confirmed these results; however, most have been retrospective [4,11,13], which is a limitation. Although the number of participants was small, pregnancy complications were confirmed in a prospective study using actual indoor PM_2.5_ measurement. 

Several studies have shown that male fetuses are vulnerable to intrauterine growth restriction [27,33] and PTB [34]. Although the sex difference in fetuses affected by PM_2.5_ remains controversial [11,16,33], this study showed an association between LBW and PM_2.5_, especially in male infants. To understand the sex differences, it is necessary to understand the biological mechanisms underlying PM_2.5_ [35]. Male fetuses grow faster than female fetuses and require more oxygen; therefore, the possibility that toxic substances inhibit this process has been suggested [33,35]. Another mechanism is that an increase in inflammatory mediators in the blood caused by air pollution increases the blood viscosity [36], which affects placental function. In general, placental dysfunction is more prevalent in male fetuses [37,38]. The cohort of the APPO study could not confirm the sex differences owing to the small number of participants, but it is necessary to confirm whether PM_2.5_ actually affects fetal growth to continuously recruit participants in the future. 

Similar to previous studies showing the relationships between PM_2.5_ and environmental pollutants and oxidative stress, this study showed a relationship between PM_2.5_ and 8-OHdG levels [39]. In brief, 8-OHdG is a reactive oxygen species that has been used as a marker of DNA damage because of its mutagenic potential [40]. The results of this study show the possibility of using 8-OHdG as a biomarker of PM_2.5_ exposure. In particular, urine samples have the advantage that they can be collected from patients non-invasively, and since 8-OHdG shows high stability in urine, it can be used as a biomarker [41]. By verifying the effectiveness of 8-OHdG in the future, it can be used as a biomarker for biomonitoring, suggesting the possibility that the use of antioxidants affects the prognosis of newborns [24]. PM not only affects the outcomes of newborns and pregnancy but also long-term diseases in offspring, according to the concept of fetal programming [42,43]. 

Most existing studies have estimated fine dust exposure concentrations based on differences in air quality by region and location [4,11,28,44]. However, as 80–90% of individuals live indoors, owing to COVID-19, the outdoor activity of pregnant women further decreased, and the importance of indoor air quality has been emphasized [45,46]. In this APPO study, indoor PM_2.5_ measurements were performed in the residences of pregnant women for at least 1 week, and it is thought that this can give causality to the biological mechanism of any relationship between PM_2.5_ and a disease. The results of this study, using correlation analysis between indoor and outdoor PM_2.5_, explain that outdoor conditions significantly affect indoor air quality; however, the correlation can be explained by only 18.7% influence. This indicates that the indoor air quality can be affected by various human activities, including smoking and cooking [14,17], thereby suggesting that education regarding lifestyle changes may also be important for better pregnancy outcomes. Through this survey, we will conduct additional research on the group in which indoor PM_2.5_ increases in preparation for actual outdoor PM_2.5_. We plan to conduct further research on active intervention measures to prevent pregnancy complications. 

In this study, there was a difference in PM_2.5_ concentrations between Cohorts I and II, either because of the air pollution reduction effect owing to the COVID-19 lockdown [47] or because the participants in Cohort I were recruited from a metropolitan area, whereas the participants in Cohort II were recruited from the countryside. 

To our knowledge, this was the first study to examine the relationship between fine dust and fetal growth in pregnant Korean women with continuously measured indoor PM_2.5_ concentrations. It was also the first study to measure oxidative stress and develop fine dust exposure evaluation indicators in pregnant women with actual indoor PM_2.5_ measurement.

The strength of this study was that it was a prospective multicenter cohort study that investigated the maternal and fetal health effects of PM on pregnancy in patients from various regions of South Korea. Compared with a previous study that measured only outdoor data, it was more reasonable to confirm the causal relationship between fine dust and pregnancy complications through fine dust concentrations measured using individual indoor air quality values. 

However, this study had several limitations. Although we measured the indoor fine dust concentrations of the participants for at least 1 week, there was a limitation in that the cumulative concentration during the entire pregnancy could not be calculated. Moreover, other stressful conditions that may affect pregnancy complications, including alcohol abuse and infection, were not considered. Recent, various studies have reported on air pollutants, including PM_1.0_, and their health effects; however, this study focused on PM_2.5_ and did not analyze other air pollutants.

A correlation between PM_2.5_ and 8-OHdG was observed, but no correlation with actual complications was found. Therefore, in the future, we will recruit more participants and make efforts to calculate the cumulative concentrations of fine dust in pregnant women and evaluate the biological mechanisms of 8-OHdG as a biomarker. Moreover, various stressful conditions that can be confounding variables in pregnancy outcomes should be included, and the impact of other air pollutants should be considered during the analysis.

## 5. Conclusions

This study confirmed the association between PM_2.5_ and LBW through a retrospective cohort study, and its effect was found to be more pronounced in the male and PTB groups. Furthermore, through a multicenter prospective cohort study, we determined the relationships between PM_2.5_ and birth height, SGA, and PTB by measuring the actual indoor PM_2.5_. The oxidative stress marker showed a positive correlation with PM_2.5_, suggesting its potential as a biomarker for PM_2.5_ exposure. However, further research on the actual mechanisms of action, efficacy, and relevance to pregnancy outcomes is needed. Furthermore, we will continue to identify interventions to lower indoor PM levels.

## Figures and Tables

**Table 1:** Characteristics of study population of Cohort I.

Characteristics	NBW (*n* = 1569)		LBW (*n* = 311)		*p*-Value
Age (years)	33.20	4.16	32.79	4.47	0.12
Pre-BMI (kg/m^2^)	21.46	3.59	21.52	3.65	0.92
GAB (wks)	39.02	1.35	33.30	3.49	<0.01 *
Neonate Sex					
Male	791	82.14%	172	17.86%	0.12
Female	780	84.78%	140	15.22%	
Birth weight (g)	3266.44	394.83	1824.04	497.55	<0.01 *
Birth height (cm)	49.76	1.88	42.08	4.06	<0.01 *
APGAR 1 min	9.30	1.12	6.76	2.54	<0.01 *
APGAR 5 min	9.88	0.55	8.27	2.24	<0.01 *
Pregnancy complications					
PTB	81	24.40%	251	75.60%	<0.01 *

Categorical variables are expressed as frequencies (percentages) and were analyzed using the chi-square test. Continuous variables are expressed as means ± SDs and were compared using *t*-tests. APGAR, appearance, pulse, grimace, activity, respiration; BMI, body mass index; GAB, gestational age at birth; SD, standard deviation. * *p* < 0.05 considered statistically significant.

**Table 2:** Summary of PM_2.5_ concentration (μg/m^3^) by second trimester of pregnancy.

	Mean	SD	Min	25th	50th	75th	Max	*p*-Value
[Cohort I]	27.35	4.48	17.73	23.33	27.89	30.72	41.68	<0.01 *^,1^
[Cohort II]								<0.01 *^,2^
Indoor	10.57	10.47	0.53	4.35	7.8	12.02	69.25	
Outdoor	17.27	7.48	1.69	12.00	16.71	20.14	43.50	

^1^ Statistical significance of concentration of PM_2.5_ between Cohorts I and II. ^2^ Statistical significance between indoor and outdoor PM_2.5_ of Cohort II. PM_2.5_, particulate matter 2.5. * *p* < 0.05 considered statistically significant.

**Table 3:** Comparison of PM_2.5_ exposure (μg/m^3^) by each trimester in NBW, LBW, NBH, and LBH, according to sex.

Exposure Period	All Newborns			Males			Females		
	NBW (*n* = 1567)	LBW (*n* = 313)	*p*-Value	NBW (*n* = 791)	LBW (*n* = 169)	*p*-Value	NBW (*n* = 774)	LBW (*n* = 141)	*p*-Value
**Entire**	27.17 ± 4.30	27.40 ± 4.67	0.43	27.24 ± 4.37	27.79 ± 4.82	0.17	27.11 ± 4.24	26.93 ± 4.48	0.65
**First trimester**	28.75 ± 7.63	28.04 ± 7.70	0.15	28.91 ± 7.87	28.38 ± 7.94	0.45	28.59 ± 7.38	27.71 ± 7.42	0.21
**Second trimester**	26.23 ± 6.78	27.21 ± 6.53	0.02 *	26.26 ± 6.70	27.89 ± 6.79	0.01 *	26.22 ± 6.86	26.38 ± 6.15	0.78
**Third trimester**	26.40 ± 6.43	27.04 ± 8.56	0.23	26.39 ± 6.28	26.94 ± 8.63	0.46	26.38 ± 6.57	27.03 ± 8.40	0.40

Continuous variables are expressed as means ± SDs and compared using *t*-tests. LBH, low birth height; LBW, low birth weight; NBH, normal birth height; NBW, normal birth weight. * *p* < 0.05 considered statistically significant.

**Table 4:** Subgroup analysis for comparison of PM_2.5_ exposure (μg/m^3^) by each trimester in the NBW and LBW at preterm in Cohort I.

Exposure Period	All Newborns			Male Infants			Female Infants		
	PNBW (*n* = 84)	PLBW (*n* = 260)	*p*-Value	PNBW (*n* = 48)	PLBW (*n* = 149)	*p*-Value	PNBW (*n* = 36)	PLBW (*n* = 111)	*p*-Value
**Entire**	25.89 ± 4.15	27.29 ± 4.78	0.02 *	25.06 ± 4.03	27.61 ± 4.90	<0.01 *	27.00 ± 4.10	26.87 ± 4.64	0.88
**First trimester**	25.81 ± 6.63	27.58 ± 7.57	0.05	24.35 ± 6.21	27.73 ± 7.65	<0.01 *	27.77 ± 6.76	27.48 ± 7.50	0.84
**Second trimester**	25.57 ± 6.26	27.25 ± 6.58	0.04 *	24.82 ± 5.87	27.81 ± 6.82	0.01 *	26.58 ± 6.69	26.51 ± 6.22	0.95
**Third trimester**	26.69 ± 6.15	27.23 ± 8.98	0.55	26.92 ± 5.61	27.27 ± 9.09	0.80	26.39 ± 6.86	27.02 ± 8.77	0.66

Continuous variables were expressed as means ± SDs and compared using *t*-test. PNBW, preterm normal birth weight; PLBW, preterm low birth weight. * *p* < 0.05 considered statistically significant.

**Table 5:** Association between PM_2.5_ exposure (per 1 μg/m^3^) in each trimester and logistic regression analysis.

Risk of LBW by PM_2.5_ Exposure (per 1 μg/m^3^)									
	All Newborns			Male Infants			Female Infants		
Stage	OR	95% CI	*p*-Value	OR	95% CI	*p*-Value	OR	95% CI	*p*-Value
Entire	1.06	1.01–1.10	0.02 *	1.12	1.04–1.20	<0.01 *	1.00	0.93–1.07	0.94
First trimester	1.02	1.00–1.05	0.08	1.05	1.01–1.10	0.02 *	1.00	0.96–1.04	0.86
Second trimester	1.03	1.00–1.06	0.04 *	1.07	1.03–1.12	<0.01 *	1.00	0.96–1.04	0.90
Third trimester	1.00	0.98–1.03	0.80	1.00	0.96–1.04	0.85	1.01	0.97–1.05	0.73

Models adjusted for maternal age, parity, maternal pre-pregnancy body mass index, preeclampsia, and gestational age. CI, confidence interval; OR, odds ratio. * *p* < 0.05 considered statistically significant.

**Table 6:** Characteristics of study population in Cohort II.

Characteristics	Low PM_2.5_ (*n* = 191)		High PM_2.5_ (*n* = 115)		*p*-Value
Age (years)	33.74	±3.62	33.12	±4.50	0.19
Pre-BMI (kg/m^2^)	21.86	±3.31	21.56	±3.17	0.43
Married state					
Married	167	100.0%	133	97.8%	0.16
Unmarried	0	0.0%	3	2.2%	
Education level					0.14
High school graduation or below	13	6.8%	13	9.5%	
University graduates	154	92.3%	123	90.4%	
Occupation					
Yes	112	67.1%	94	69.1%	0.70
No	55	32.9%	42	30.9%	
Monthly income					
<4 million won	35	36.1%	35	37.6%	0.95
4–6 million won	27	27.8%	24	25.8%	
>6 million won	35	36.1%	34	36.6%	
Gravidity					
1	80	47.9%	68	50.0%	0.65
2	61	36.5%	50	36.8%	
≥3	26	15.6%	18	13.2%	
Pregnancy methods					
Natural	143	85.6%	116	85.3%	0.91
IUI	2	1.2%	1	0.7%	
IVF-ET	22	13.2%	19	14.0%	

Categorical variables are expressed as frequencies (percentages) and were analyzed using chi-square and Fisher’s exact tests. Continuous variables are expressed as means ± SDs and were compared using the *t*-test or Mann–Whitney U test. IUI, intrauterine insemination; IVF-ET, in vitro fertilization–embryo transfer.

**Table 7:** Oxidative stress and inflammatory markers between the low and high PM_2.5_ groups.

	Low PM_2.5_ (*n* = 191)		High PM_2.5_ (*n* = 115)		*p*-Value
Oxidative stress marker					
8-OHdG (ng/mL)	92.20	61.16–138.19	113.55	79.69–153.27	0.02 *
MDA (μM)	18.49	8.66–37.18	16.02	9.99–30.34	0.54
Inflammatory marker					
Hs-CRP (mg/L)	2.00	1.00–3.00	1.79	1.00–2.99	0.74
WBC (1 × 10^3^/μL)	8.80	7.00–9.72	8.00	7.00–10.00	0.98

Continuous variables are expressed as medians (interquartile ranges) and were compared using the Mann–Whitney U test. 8-OHdG, 8-hydroxy-2-deoxyguanosine; Hs-CRP, high-sensitivity C-reactive protein; MDA, malondialdehyde; WBC, white blood cells. * *p* < 0.05 considered statistically significant.

**Table 8:** Association between PM_2.5_ exposure and pregnancy complications.

	Low PM_2.5_ (*n* = 191)		High PM_2.5_ (*n* = 115)		*p*-Value
Pregnancy outcome					
GAB (wks)	38.22	±1.59	37.96	±1.95	0.22
Delivery mode					0.86
ND	61	36.5%	51	37.5%	
CS	106	63.5%	85	62.5%	
Neonate Sex					
Male	112	58.9%	56	49.1%	0.10
Female	78	41.1%	58	50.9%	
Birth weight (g)	3150.7	±391.6	3112.0	±522.5	0.50
Birth height (cm)	49.5	±2.2	48.9	±2.8	0.04 *
APGAR 1 min	8.45	±1.22	8.36	±	0.61
APGAR 5 min	9.43	±0.84	9.21	±1.37	0.13
Pregnancy complications					
LBW	4	5.1%	4	6.9%	0.47
LBH	5	2.6%	8	7.0%	0.07
PTB	8	4.2%	15	13.2%	<0.01 *
SGA	1	0.6%	7	5.1%	0.01 *
NICU admission	20	12.0%	23	16.9%	0.32

Categorical variables are expressed as frequencies (percentages) and were analyzed using chi-square and Fisher’s exact tests. Continuous variables are expressed as means ± SDs or medians (interquartile range) and were compared using the *t*-test or Mann–Whitney U test. CS, cesarean section; ND, normal delivery; NICU, neonatal intensive care unit; SGA, small for gestational age. * *p* < 0.05 considered statistically significant.

## Data Availability

The data supporting the findings of this study are available in the article. Raw data supporting the findings of this study cannot be shared openly to protect personally identifiable information.

## Supplementary Materials

The following supporting information can be downloaded at: https://www.mdpi.com/article/10.3390/antiox12111916/s1, Table S1: Summary of PM_2.5_ concentration (μg/m^3^) by each trimester of pregnancy of Cohort I; Table S2: Subgroup analysis of comparison of PM_2.5_ exposure (μg/m^3^) by each trimester in the NBW and LBW at full term of Cohort I.

## References

[1] Park S., Moon J., Kang N., Kim Y.H., You Y.A., Kwon E., Ansari A., Hur Y.M., Park T., Kim Y.J. (2022). Predicting preterm birth through vaginal microbiota, cervical length, and WBC using a machine learning model. Front. Microbiol..

[2] Goldenberg R.L., Culhane J.F., Iams J.D., Romero R. (2008). Epidemiology and causes of preterm birth. Lancet.

[3] Yang T., Chen R., Gu X., Xu J., Yang L., Zhao J., Zhang X., Bai C., Kang J., Ran P. (2021). Association of fine particulate matter air pollution and its constituents with lung function: The China Pulmonary Health study. Environ. Int..

[4] Shen Y., Wang C., Yu G., Meng X., Wang W., Kan H., Zhang J., Cai J. (2022). Associations of Ambient Fine Particulate Matter and Its Chemical Constituents with Birth Weight for Gestational Age in China: A Nationwide Survey. Environ. Sci. Technol..

[5] Gray S.C., Edwards S.E., Miranda M.L. (2010). Assessing exposure metrics for PM and birth weight models. J. Expo. Sci. Environ. Epidemiol..

[6] Percy Z., DeFranco E., Xu F., Hall E.S., Haynes E.N., Jones D., Muglia L.J., Chen A. (2019). Trimester specific PM_2.5_ exposure and fetal growth in Ohio, 2007–2010. Environ. Res..

[7] Kloog I., Melly S.J., Ridgway W.L., Coull B.A., Schwartz J. (2012). Using new satellite based exposure methods to study the association between pregnancy PM_2.5_ exposure, premature birth and birth weight in Massachusetts. Environ. Health.

[8] Han Z., Lutsiv O., Mulla S., Rosen A., Beyene J., McDonald S.D. (2011). Low gestational weight gain and the risk of preterm birth and low birthweight: A systematic review and meta-analyses. Acta Obstet. Gynecol. Scand..

[9] Salihu H.M., Garcia B.Y., Dongarwar D., Maiyegun S.O., Yusuf K.K., Agili D.E.A. (2021). Maternal pre-pregnancy underweight and the risk of small-for-gestational-age in Asian-American ethnic groups. Obstet. Gynecol. Sci..

[10] Valero De Bernabé J., Soriano T., Albaladejo R., Juarranz M., Calle M.E., Martínez D., Domínguez-Rojas V. (2004). Risk factors for low birth weight: A review. Eur. J. Obstet. Gynecol. Reprod. Biol..

[11] Bachwenkizi J., Liu C., Meng X., Zhang L., Wang W., van Donkelaar A., Martin R.V., Hammer M.S., Chen R., Kan H. (2022). Maternal exposure to fine particulate matter and preterm birth and low birth weight in Africa. Environ. Int..

[12] Stieb D.M., Chen L., Eshoul M., Judek S. (2012). Ambient air pollution, birth weight and preterm birth: A systematic review and meta-analysis. Environ. Res..

[13] Guo P., Chen Y., Wu H., Zeng J., Zeng Z., Li W., Zhang Q., Huo X., Feng W., Lin J. (2020). Ambient air pollution and markers of fetal growth: A retrospective population-based cohort study of 2.57 million term singleton births in China. Environ. Int..

[14] Zhang L., Ou C., Magana-Arachchi D., Vithanage M., Vanka K.S., Palanisami T., Masakorala K., Wijesekara H., Yan Y., Bolan N. (2021). Indoor Particulate Matter in Urban Households: Sources, Pathways, Characteristics, Health Effects, and Exposure Mitigation. Int. J. Environ. Res. Public Health.

[15] Shezi B., Jafta N., Naidoo R.N. (2020). Exposure assessment of indoor particulate matter during pregnancy: A narrative review of the literature. Rev. Environ. Health.

[16] Cho H.J., Lee S.H., Lee S.Y., Kim H.C., Kim H.B., Park M.J., Yoon J., Jung S., Yang S.I., Lee E. (2021). Mid-pregnancy PM_2.5_ exposure affects sex-specific growth trajectories via ARRDC3 methylation. Environ. Res..

[17] Patelarou E., Kelly F.J. (2014). Indoor exposure and adverse birth outcomes related to fetal growth, miscarriage and prematurity-a systematic review. Int. J. Environ. Res. Public Health.

[18] Kim J.Y., Lee E.Y., Choi I., Kim J., Cho K.H. (2015). Effects of the Particulate Matter_2.5_ (PM_2.5_) on Lipoprotein Metabolism, Uptake and Degradation, and Embryo Toxicity. Mol. Cells.

[19] Nääv Å., Erlandsson L., Isaxon C., Åsander Frostner E., Ehinger J., Sporre M.K., Krais A.M., Strandberg B., Lundh T., Elmér E. (2020). Urban PM2.5 Induces Cellular Toxicity, Hormone Dysregulation, Oxidative Damage, Inflammation, and Mitochondrial Interference in the HRT8 Trophoblast Cell Line. Front. Endocrinol..

[20] Rosa M.J., Hsu H.L., Just A.C., Brennan K.J., Bloomquist T., Kloog I., Pantic I., Mercado García A., Wilson A., Coull B.A. (2019). Association between prenatal particulate air pollution exposure and telomere length in cord blood: Effect modification by fetal sex. Environ. Res..

[21] Liu X.C., Strodl E., Wu C.A., Huang L.H., Yin X.N., Wen G.M., Sun D.L., Xian D.X., Chen W.Q. (2022). Critical window for the association between prenatal environmental tobacco smoke exposure and preterm birth. Environ. Res..

[22] Chen W.J., Rector A.M., Guxens M., Iniguez C., Swartz M.D., Symanski E., Ibarluzea J., Ambros A., Estarlich M., Lertxundi A. (2022). Susceptible windows of exposure to fine particulate matter and fetal growth trajectories in the Spanish INMA (INfancia y Medio Ambiente) birth cohort. Environ. Res..

[23] Guo C., Lv S., Liu Y., Li Y. (2022). Biomarkers for the adverse effects on respiratory system health associated with atmospheric particulate matter exposure. J. Hazard. Mater..

[24] Lee A.G., Cowell W., Kannan S., Ganguri H.B., Nentin F., Wilson A., Coull B.A., Wright R.O., Baccarelli A., Bollati V. (2020). Prenatal particulate air pollution and newborn telomere length: Effect modification by maternal antioxidant intakes and infant sex. Environ. Res..

[25] Kim Y.J., Hong Y.C., Lee K.H., Park H.J., Park E.A., Moon H.S., Ha E.H. (2005). Oxidative stress in pregnant women and birth weight reduction. Reprod. Toxicol..

[26] Shastri L., Pammal R.S., Mani I., Thomas T., Kurpad A.V. (2016). Oxidative stress during early pregnancy and birth outcomes. Public. Health Nutr..

[27] Quraishi S.M., Hazlehurst M.F., Loftus C.T., Nguyen R.H.N., Barrett E.S., Kaufman J.D., Bush N.R., Karr C.J., LeWinn K.Z., Sathyanarayana S. (2022). Association of prenatal exposure to ambient air pollution with adverse birth outcomes and effect modification by socioeconomic factors. Environ. Res..

[28] Liang Z., Yang Y., Qian Z., Ruan Z., Chang J., Vaughn M.G., Zhao Q., Lin H. (2019). Ambient PM_2.5_ and birth outcomes: Estimating the association and attributable risk using a birth cohort study in nine Chinese cities. Environ. Int..

[29] Klepac P., Locatelli I., Korošec S., Künzli N., Kukec A. (2018). Ambient air pollution and pregnancy outcomes: A comprehensive review and identification of environmental public health challenges. Environ. Res..

[30] Yang S., Tan Y., Mei H., Wang F., Li N., Zhao J., Zhang Y., Qian Z., Chang J.J., Syberg K.M. (2018). Ambient air pollution the risk of stillbirth: A prospective birth cohort study in Wuhan, China. Int. J. Hyg. Environ. Health.

[31] Kannan S., Misra D.P., Dvonch J.T., Krishnakumar A. (2006). Exposures to airborne particulate matter and adverse perinatal outcomes: A biologically plausible mechanistic framework for exploring potential effect modification by nutrition. Environ. Health Perspect..

[32] Bekkar B., Pacheco S., Basu R., DeNicola N. (2020). Association of Air Pollution and Heat Exposure With Preterm Birth, Low Birth Weight, and Stillbirth in the US: A Systematic Review. JAMA Netw. Open.

[33] Jedrychowski W., Perera F., Mrozek-Budzyn D., Mroz E., Flak E., Spengler J.D., Edwards S., Jacek R., Kaim I., Skolicki Z. (2009). Gender differences in fetal growth of newborns exposed prenatally to airborne fine particulate matter. Environ. Res..

[34] Challis J., Newnham J., Petraglia F., Yeganegi M., Bocking A. (2013). Fetal sex and preterm birth. Placenta.

[35] Bukowski R., Smith G.C., Malone F.D., Ball R.H., Nyberg D.A., Comstock C.H., Hankins G.D., Berkowitz R.L., Gross S.J., Dugoff L. (2007). Human sexual size dimorphism in early pregnancy. Am. J. Epidemiol..

[36] Peters A., Döring A., Wichmann H.E., Koenig W. (1997). Increased plasma viscosity during an air pollution episode: A link to mortality?. Lancet.

[37] Edwards A., Megens A., Peek M., Wallace E.M. (2000). Sexual origins of placental dysfunction. Lancet.

[38] Ghidini A., Salafia C.M. (2005). Gender differences of placental dysfunction in severe prematurity. Bjog.

[39] Hu W., Wang Y., Wang T., Ji Q., Jia Q., Meng T., Ma S., Zhang Z., Li Y., Chen R. (2021). Ambient particulate matter compositions and increased oxidative stress: Exposure-response analysis among high-level exposed population. Environ. Int..

[40] Pilger A., Rüdiger H.W. (2006). 8-Hydroxy-2′-deoxyguanosine as a marker of oxidative DNA damage related to occupational and environmental exposures. Int. Arch. Occup. Environ. Health.

[41] Poulsen H.E., Loft S., Prieme H., Vistisen K., Lykkesfeldt J., Nyyssonen K., Salonen J.T. (1998). Oxidative DNA damage in vivo: Relationship to age, plasma antioxidants, drug metabolism, glutathione-S-transferase activity and urinary creatinine excretion. Free Radic. Res..

[42] Li S., Liu Y., Liu B., Hu Y.Q., Ding Y.Q., Zhang J., Feng L. (2022). Maternal urban particulate matter exposure and signaling pathways in fetal brains and neurobehavioral development in offspring. Toxicology.

[43] Öztürk H.N.O., Türker P.F. (2021). Fetal programming: Could intrauterin life affect health status in adulthood?. Obstet. Gynecol. Sci..

[44] Yuan L., Zhang Y., Wang W., Chen R., Liu Y., Liu C., Kan H., Gao Y., Tian Y. (2020). Critical windows for maternal fine particulate matter exposure and adverse birth outcomes: The Shanghai birth cohort study. Chemosphere.

[45] Li Z., Wen Q., Zhang R. (2017). Sources, health effects and control strategies of indoor fine particulate matter (PM_2.5_): A review. Sci. Total Environ..

[46] Park S., Marcotte R.T., Staudenmayer J.W., Strath S.J., Freedson P.S., Chasan-Taber L. (2022). The impact of the COVID-19 pandemic on physical activity and sedentary behavior during pregnancy: A prospective study. BMC Pregnancy Childbirth.

[47] Ju M.J., Oh J., Choi Y.H. (2021). Changes in air pollution levels after COVID-19 outbreak in Korea. Sci. Total Environ..

