# Correction: Park et al. Effect of Particulate Matter 2.5 on Fetal Growth in Male and Preterm Infants through Oxidative Stress. *Antioxidants* 2023, *12*, 1916

**DOI:** 10.3390/antiox13020135

**Published:** 2024-01-23

**Authors:** Sunwha Park, Eunjin Kwon, Gain Lee, Young-Ah You, Soo Min Kim, Young Min Hur, Sooyoung Jung, Yongho Jee, Mi Hye Park, Sung Hun Na, Young-Han Kim, Geum Joon Cho, Jin-Gon Bae, Soo-Jeong Lee, Sun Hwa Lee, Young Ju Kim

**Affiliations:** 1 Department of Obstetrics and Gynecology, College of Medicine, Ewha Womans University, Seoul 07985, Republic of Korea; clarrissa15@gmail.com (S.P.); yerang02@naver.com (Y.-A.Y.); k0507hym@hanmail.net (Y.M.H.); jsmed9006@naver.com (S.J.); 2 Division of Allergy and Respiratory Disease Research, Department of Chronic Disease Convergence Research, Korea National Institute of Health, Cheongju-si 28159, Republic of Korea; friendkej1004@hanmail.net; 3 Graduate Program in System Health Science and Engineering, Ewha Womans University, Seoul 07985, Republic of Korea; loveleee0102@gmail.com (G.L.); soomnium@naver.com (S.M.K.); 4 Advanced Biomedical Research Institute, Ewha Womans University Seoul Hospital, Seoul 07804, Republic of Korea; jyongho@ewha.ac.kr; 5 Department of Obstetrics and Gynecology, Ewha Womans University Seoul Hospital, Seoul 07804, Republic of Korea; ewhapmh@ewha.ac.kr; 6 Department of Obstetrics and Gynecology, School of Medicine, Kangwon National University, Chuncheon-si 24289, Republic of Korea; lahun@kangwon.ac.kr; 7 Department of Obstetrics and Gynecology, College of Medicine, Yonsei University, Seoul 03722, Republic of Korea; yhkim522@yuhs.ac; 8 Department of Obstetrics and Gynecology, College of Medicine, Korea University, Seoul 02841, Republic of Korea; geumjoon@korea.ac.kr; 9 Department of Obstetrics and Gynecology, School of Medicine, Keimyung University, Dongsan Medical Center, Daegu 42601, Republic of Korea; gonmd@dsmc.or.kr; 10 Department of Obstetrics and Gynecology, College of Medicine, Ulsan University, Ulsan 44610, Republic of Korea; exsjlee@uuh.ulsan.kr; 11 Seegene Medical Foundation, Seoul 04805, Republic of Korea; lshkim@neolab.co.kr

In the original publication [1], there was a mistake in Figure 3 as published. The contents of Figure 3 and the numbers in Table 8 should be the same, but the wrong figure was used before. The correct Figure 3 appears below. The authors state that the scientific conclusions are unaffected. This correction was approved by the Academic Editor. The original publication has also been updated.

## Figures and Tables

**Figure 3:**
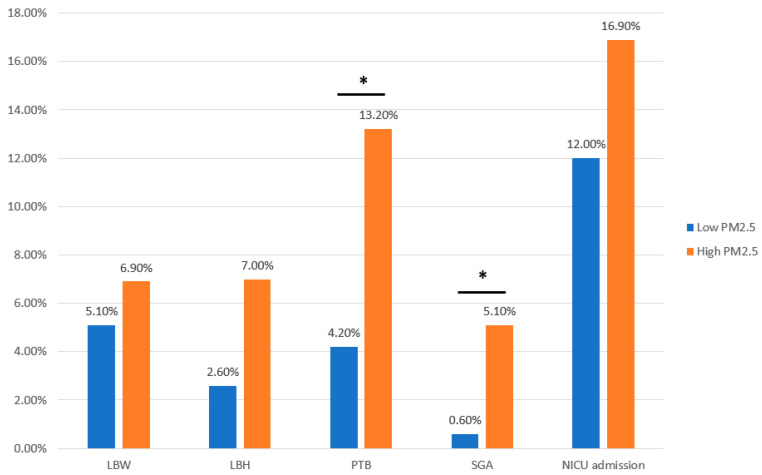
Pregnancy complications in the PM_2.5_ concentration group. LBW, low birth weight; LBH, low birth height; PTB, preterm birth; SGA, small for gestational age; NICU, neonatal intensive care unit. * *p* < 0.05 considered statistically significant.
