# Supplementary material for: Effect of Particulate Matter 2.5 on Fetal Growth in Male and Preterm Infants through Oxidative Stress
Source: Antioxidants (Basel). 2023 Oct 26;12(11):1916. doi: 10.3390/antiox12111916 (PMC10669397; doi:10.3390/antiox12111916)
Supplement: Supplementary file 1 [file antioxidants-12-01916-s001.zip › antioxidants-2646911-supplementary.pdf]

Supplementary

Supplementary Table S1. Summary of $PM_{2.5}$ concentration (ug/m^3) by each trimester of pregnancy of Cohort I.

Exposure period	Mean	SD	Min	25th	50th	75th	Max
Entire	27.35	4.48	17.73	23.33	27.89	30.72	41.68
1st trimester	28.76	7.86	14.75	21.79	27.29	35.93	50.91
2nd trimester	26.59	6.77	13.74	20.99	26.20	32.22	44.74
3rd trimester	26.55	6.73	13.02	21.91	25.86	31.15	90.58

Supplementary Table S2. Subgroup analysis of comparison of $PM_{2.5}$ exposure (ug/m^3) by each trimester in the NBW and LBW at full term of cohort I.

**Exposure period**	**All newborns**			**Boys**			**Girls**		
	TNBW (n=1485)	TLBW (n=51)	*p-Value*	TNBW (n=743)	TLBW (n=22)	*p-Value*	TNBW (n=742)	TLBW (n=29)	*p-Value*
Entire	27.25 ± 4.30	27.88 ± 4.12	0.30	27.38 ± 4.36	28.79 ± 4.26	0.13	27.11 ± 4.25	27.14 ± 3.91	0.97
1st trimester	28.91 ± 7.65	30.09 ± 8.00	0.28	29.20 ± 7.88	31.95 ± 8.72	0.10	28.63 ± 7.41	28.56 ± 7.15	0.96
2nd trimester	26.27 ± 6.80	27.00 ± 6.37	0.45	26.35 ± 6.74	28.32 ± 6.72	0.17	26.20 ± 6.87	25.92 ± 5.97	0.83
3rd trimester	26.38 ± 6.44	26.19 ± 6.30	0.83	26.35 ± 6.32	25.11 ± 5.30	0.35	26.38 ± 6.56	27.07 ± 6.99	0.59

Continuous variables were expressed as mean ± standard deviation (SD) and were compared using the *t*-test. TNBW, term normal birth weight; TLBW, term low birth weight
